# Antifungal Peptides of the AFP Family Revisited: Are These Cannibal Toxins?

**DOI:** 10.3390/microorganisms6020050

**Published:** 2018-06-02

**Authors:** Vera Meyer, Sascha Jung

**Affiliations:** Department Applied and Molecular Microbiology, Technische Universität Berlin, Institute of Biotechnology, Gustav-Meyer-Allee 25, D-13355 Berlin, Germany; s.jung@tu-berlin.de

**Keywords:** antimicrobial peptide, antifungal, AFP, AnAFP, mode of action, *Aspergillus niger*, *Aspergillus giganteus*, sporulation, *Bacillus*, cannibal toxin

## Abstract

The emergence and spread of pathogenic fungi resistant to currently used antifungal drugs represents a serious challenge for medicine and agriculture. The use of smart antimicrobials, so-called “dirty drugs” which affect multiple cellular targets, is one strategy to prevent resistance. Of special interest is the exploitation of the AFP family of antimicrobial peptides, which include its founding member AFP from *Aspergillus giganteus*. This latter is a highly potent inhibitor of chitin synthesis and affects plasma membrane integrity in many human and plant pathogenic fungi. A transcriptomic meta-analysis of the *afp-*encoding genes in *A. giganteus* and *A. niger* predicts a role for these proteins during asexual sporulation, autophagy, and nutrient recycling, suggesting that AFPs are molecules important for the survival of *A. niger* and *A. giganteus* under nutrient limitation. In this review, we discuss parallels which exist between AFPs and bacterial cannibal toxins and provide arguments that the primary function of AFPs could be to kill genetically identical siblings. We hope that this review inspires computational and experimental biologists studying alternative explanations for the nature and function of antimicrobial peptides beyond the general assumption that they are mere defense molecules to fight competitors.

## 1. Introduction

The antimicrobial peptide field is rapidly moving forward. PubMed lists about 9000 publications for the year 2017 alone, and the Antimicrobial Peptide Database (APD, [1]) currently contains data on 2950 antimicrobial peptides (AMPs). The majority of currently studied AMPs are of mammalian origin (~75%), followed by plant (~13%) and bacterial AMPs (~10%). Only 1% of the currently known and studied AMPs are from fungi. Although AMPs are produced in phylogenetically very distant domains and kingdoms, they display a remarkable degree of structural and functional conservation. Unifying structural characteristics include high stability due to intramolecular disulfide bridge formation, predominant β-sheet formation, a net cationic charge, an amphipathic surface, high membrane activity, and the presence of a γ-core motif thought to mediate membrane interaction [2,3].

**Figure 1:**
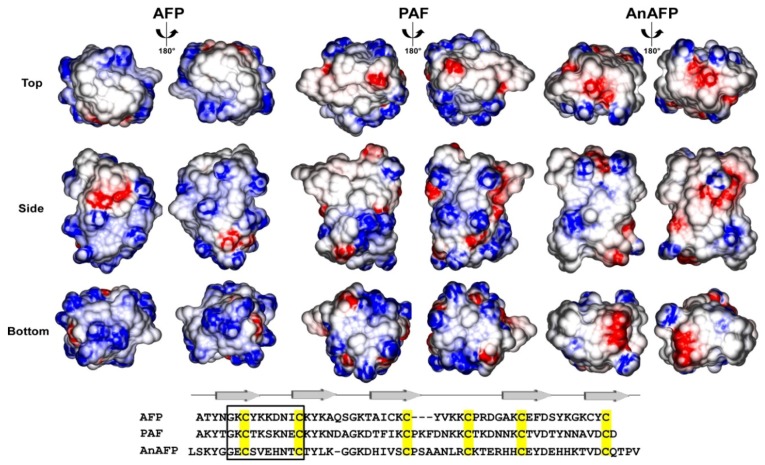
Electrostatic surface potentials of AFP, PAF, and AnAFP. AFP and PAF were derived from PDB accession codes 1AFP or 1KCN, respectively, whereas the structure of AnAFP was generated by molecular modeling using the structure of AFP as the template. Negatively charged regions are colored red, positively charged ones blue, and uncharged ones white. Graphical representations displaying top, side, and bottom views of the peptides were generated using the program GRASP2 [11]. An alignment of AFP, PAF, and AnAFP is given below. The box depicts residues of the γ-core motif. Arrows on top represent beta-strands.

In the filamentous fungal community, Cys-stabilized antimicrobial peptides from filamentous fungi are of special interest because many of these peptides display efficient antifungal properties in the micromolar range without negatively interfering with the viability of bacterial, plant, or mammalian cells [4,5,6,7]. Historically, the focus has been on two peptides—AFP from *Aspergillus giganteus* and PAF from *Penicillium chrysogenum*—as these peptides were the first studied and considered as interesting lead compounds for the development of novel antifungal drugs to combat against devastating fungal pathogens threatening human welfare and food security [5,8]. Both AFP and PAF are members of the AFP family (named after its founder member AFP, isolated for the first time in 1965 from the culture supernatant of *Aspergillus giganteus* [9]), which currently comprises about 50 peptides [10]. All orthologs identified so far are encoded in 35 *Ascomycetes* species and include *Aspergillus*, *Beauveria bassiana*, *Botryotinia fuckeliana*, *Colletotrichum orbiculare*, *Diplodia seriata*, *Epichloe festucae*, *Fusarium* spp., *Gibberella zeae* (teleomorph of *F*. *graminearum)*, *Monascus pilosus*, *Ophiocordyceps unilateralis, Penicillium*, and *Pyrenophora* spp. [10]. All peptides display the cysteine-spacing pattern CX_(6)_CX_(11–12)_CX_(4–9)_CX_6_CX_10–13_C present in AFP and PAF and are β-strand proteins possessing a γ-core motif (Figure 1), except for the AFP ortholog of *P*. *oxicalum* that lacks a γ-core motif [10].

The last 10 years have witnessed an increasing interest in antifungal peptides of fungal origin. Their mode of action on fungal strains including model strains and human and plant pathogens was not only studied for AFP and PAF, but for many more peptides belonging to the AFP family, including AnAFP from *A. niger*, NAFP from *N. fischeri*, PAFB from *P. chrysogenum*, and AFPB from *P. digitatum* [10,12,13,14]. In this review, we discuss the current knowledge on their expression and mode of actions. We stress, however, that the review is meant to be representative and not comprehensive and aims to a radical change of perspective: a change from a purely applied perspective on AFPs to a nonanthropocentric view on these molecules. We are convinced that the narrow human conception of AFPs (and in general AMPs) as bioactive molecules valued for antifungal (antimicrobial) therapeutic applications completely neglects their role in controlling different biological processes in their producing organisms. With this review, we thus want to broaden the general conception of these peptides. We show that parallels exist to cannibal toxins in bacteria, and we will provide plausible arguments that AFPs are similarly important molecules for their hosts to ensure the survival of a subpopulation of its producing organism and, by this, survival of the whole species.

## 2. Antifungal Modes of Action of AFPs: Similar, but Not the Same

Although the *afp* gene from *A. giganteus* was already identified 25 years ago [15], and despite its huge application potential, only 35 publications studying the molecular mechanisms behind its expression and the mode of action of AFP are listed in PubMed. The research community studying AMPs is huge; however, scientists are largely oblivious to research on AMPs of fungal origin, although this might provide insights into the general function of AMPs from other kingdoms or even domains. In brief, AFP is a 51-amino acid, cysteine-rich, 5.8 kDa amphipathic peptide with a positive net charge. *A. giganteus* secretes AFP into the surrounding medium under nonfavorable growth conditions including carbon starvation, heat shock, and pH stress [16,17]. It binds to the cell wall and plasma membrane of sensitive filamentous fungi, where it induces loss of plasma membrane integrity and eventually causes membrane permeabilization [18,19,20]. AFP has been shown to inhibit chitin synthase activity in sensitive filamentous fungi, which is thought to be mediated via its chitin-binding region [18]. AFP can also be found intracellularly in collapsed and dead cells of sensitive fungi [19,20], where it might bind to anionic molecules such as DNA and RNA via its oligonucleotide/oligosaccharide-binding (OB) fold [21]. Notably, filamentous fungi differ in their susceptibility towards AFP. Some species are highly sensitive (minimal inhibitory concentration (MIC): 0.1–10 μg/mL; e.g., *A. niger*, *F. oxysporum*), others are moderately sensitive (100 < MIC < 400 μg/ml; e.g., *A. giganteus*), and some are even resistant (e.g., *P. chrysogenum*). We could show that AFP treatment of *A. niger* (AFP-susceptible) provoked clear ultrastructural aberrations of *A. niger* cells, which are absent in the AFP-resistant strain *P. chrysogenum* [19].

One fungal defense mechanism to counteract AFP inhibitory effects is induction of the highly conserved cell wall integrity (CWI) signaling pathway, whose function is to ensure cell surface protection during cell wall stress [22,23,24,25]. AFP-mediated induction of the CWI pathway in *A. niger* thus results in higher glucan synthesis due to increased expression of the α-1,3-glucan synthase-encoding gene *agsA* [18]. However, then, if the function of the CWI pathway is meant to ensure survival of *A. niger* during the presence of AFP, why does it get killed by AFP? One explanation for this puzzling observation, which we could prove, is that induction of CWI pathway is insufficient to protect fungi against the inhibitory effects of AFP [26]. Analysis of various AFP-sensitive and AFP-resistant fungal strains indeed showed that AFP resistance is linked to upregulated chitin synthesis. However, this is mainly mediated by the calcium/calcineurin/Crz1p signaling pathway [26]. Such a defense strategy is not observed in AFP-sensitive fungi. It seems that these fungi make the wrong decision and activate the classical CWI pathway as their main defense mechanism. This pathway simply fails to counteract AFP as its output, increased glucan synthesis, does not protect against AFP [18]. In support, the AFP-related protein AFPNN5353 (which differs from AFP by only 5 amino acids) and AnAFP do also elicit the CWI pathway and to a certain extent calcium signaling in *A. niger* and *A. nidulans*, respectively ([27] and [28]). Still, it is not known why calcium signaling is only weakly activated in AFP-sensitive fungi. 

Even though AFP and PAF are very similar in their structure and antifungal spectrum, their modes of action are significantly different. PAF elicits heterotrimeric G-protein and cAMP/protein kinase A signaling in PAF-sensitive *A. nidulans*, but not the CWI pathway [27,29,30]. It hyperpolarizes the plasma membrane of sensitive fungi [30,31,32] and provokes a rapid calcium influx, followed by a sustained perturbation of calcium homeostasis [27,31]. This in turn triggers apoptosis, as reflected by the detection of increased levels of reactive oxygen species and apoptotic markers [30]. Interestingly, addition of calcium to the growth medium decreases susceptibility of aspergilli to both PAF and AFP and counteracts perturbations of intracellular calcium resting levels [26,27,31]. It was recently proven in *A. nidulans* that induction of cAMP/PKA signaling and the sustained increase of intracellular calcium levels in response to PAF treatment are linked to each other and control PAF toxicity [33]. Most recently, insights into the mode of action of a novel representative of the AFP family were published: NAFP from *Neosartorya fischeri*. In *A. nidulans*, NAFP induces apoptosis and, like PAF, elicits heterotrimeric G-protein and cAMP/protein kinase A signaling, but not the CWI pathway [14].

Remarkably, all producing strains are only moderately sensitive towards their own antifungal protein, but very sensitive towards AFPs from other filamentous fungi [10,20,34]. This implicates that they might utilize innate sensing or defense systems, enabling them to distinguish between own and alien antifungal peptides.

## 3. Gene Regulation of AFP-Encoding Genes: From the General to the Particular

**Figure 2:**
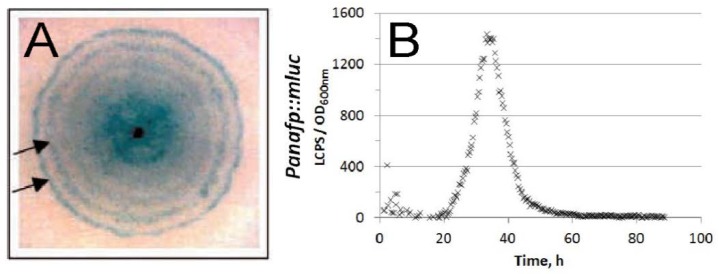
Expression of genes encoding antifungal peptides in aspergilli are under tight time-dependent control: (**A**) Oscillating expression of the *afp* gene as visualized in a 6-day old colony of an *A. giganteus* reporter strain. Here, the reporter gene β-glucuronidase (*uidA*) was put under control of the *afp* promoter. Induction of P*afp::uidA* reporter expression results in blue color formation on agar plates in a circadian manner (indicated by arrows). Blue color formation is visible only in the vegetative medium and occurs when *A. giganteus* vegetative hyphae achieve the competence to form aerial hyphae/conidiophores. Picture is reproduced from [17] with permission from Springer Nature. (**B**) Luciferase expression under control of the *anafp* promoter was measured using the reporter strain PK2.9 (P*anafp::luc*). Reporter activity was measured as luminescent counts per second normalized to culture optical density during 4 days of submerged cultivation of strain PK2.9 in microtiter plates. Picture is taken from [10], licensed under CC-BY 4.0. LCPS, luminescent counts per second.

Data on transcriptional regulation of antifungal peptides is available for only a few members of the AFP family: AFP (*A. giganteus*), PAF (*P. chrysogenum*), AnAFP (*A. niger*), and AFPB (*P. digitatum*). We and others could show that AFP, AnAFP, and PAF exhibit a temporal and spatial regulation in their native hosts and seem to be exclusively expressed in the vegetative mycelium during the developmental stage; that is, when competence for conidiophore formation is acquired [10,17,29] (see Figure 2). Notably, almost no expression of *afp*, *anafp*, or *paf* can be observed in conidiophores or conidia. Whereas the *afp* and *anafp* genes seem to be under control of the asexual developmental regulator StuA [5,10], deletion of the *paf* gene in *P. chrysogenum* is accompanied by transcriptional downregulation of the asexual developmental regulator BrlA. Concomitantly, spore production is severely reduced in a Δ*paf* strain of *P. chrysogenum* compared to the wild type [29]. These observations suggest that expression of antifungal peptides is connected with asexual development. In agreement with this, we could show that transcription of the *afp* and *anafp* genes in submerged *A. giganteus* and *A. niger* cultures, respectively, are strongly induced when the mycelium becomes subjected to carbon starvation [10,17], a condition that precedes sporulation of *Aspergillus* growing on solid media [35]. Furthermore, increased *afp* and *anafp* transcript levels can be detected during environmental stress conditions, pointing towards a defense-related function of these peptides [10,17,36]. Notably, constitutive expression of the *afpB* gene in its host disturbs vegetative growth and hyphal morphology of *P. digitatum* [37], suggesting that expression of AFPs must be tightly regulated to prevent detrimental effects if prematurely and/or overexpressed.

The ecological advantage of expressing antifungal peptides remains a mystery. If their biological function is to provide protection against other fungal inhabitants in the same ecological niche, then why do *afp, anafp*, and *paf* expression start only after depletion of carbon; that is, when it would be too late to secure carbon for survival? Similarly puzzling is the fact that the level of secreted peptides is very low and that cocultivation of *A. giganteus* with highly AFP-sensitive strains such as *F. oxysporum* or *A. niger* does not kill them [17]. Could it be that AFPs have a biological function that goes beyond antifungal activity?

### 3.1. The anafp Gene as a Paradigmatic Example

An outstanding opportunity to study the biological function(s) of antifungal peptides for their producing fungus is to scrutinize the growing body of system biology data. The antifungal peptide AnAFP from *A. niger* represents an excellent model system for several reasons: Firstly, the genome of *A. niger* has been sequenced [38], and hundreds of transcriptomics and proteomics data are publicly available for the *A. niger* strain CBS 513.88 and its derivatives [39,40] (note that the genome of *A. giganteus* has not been sequenced yet). Secondly, various bioinformatics pipelines for the analysis of genomic and transcriptomic data are available for *A. niger* [41,42]. Thirdly, our in-house *A. niger* transcriptomic database encompasses genome-wide expression profiles for a total of 155 different cultivation conditions and includes data on various nutrient sources, developmental stages, stress conditions, and cocultivations [10]. This database constitutes an invaluable treasure chest allowing studying of the cellular functions of *anafp* in a system-wide manner and to prove or disprove hypotheses regarding its biological role. Using *anafp* expression data from *A. niger* under these 155 cultivation conditions, we have recently published a meta-analysis [10], the result of which is briefly summarized as follows:

- the choice of carbon or nitrogen source does not impact *anafp* expression;
- carbon limitation and starvation strongly stimulate *anafp* expression, suggesting that *anafp* is under control of the carbon catabolite repressor CreA;
- the expression profile of *anafp* is similar to the expression profile of the early starvation response genes *atg1* (predicted Ser/Thr kinase involved in autophagy) and *nagA* (predicted autolytic β-1,6-*N*-acetylglucosaminidase);
- *anafp*, *nagA*, and *atg1* expression peaked at 16 h after carbon depletion and gradually decreased at 60 h and 140 h post-carbon depletion in submerged batch cultures. The expression peaks were paralleled with the appearance of a second hyphal population with reduced hyphal diameters (1 µm in diameter instead of 3 µm);
- under severe carbon and energy limitation resulting in very low growth rates (about 0.005 h^−1^), additionally to *anafp*, *nagA*, and *atg1*, other genes involved in nutrient mobilization, autophagy, N-acetylglucosamine metabolism, and carbohydrate transport are strongly upregulated;
- *anafp* transcript levels are low in dormant conidia and young germlings, but increase about 15-fold and 60-fold in aerial hyphae and the vegetative mycelium, respectively. This expression profile is similar to those of genes encoding chitin-remodeling enzymes (*ctcB*, *cfcI*, and *nagA*);
- in *A. niger* wild-type colonies, *anafp* expression is highest in the center of a colony and gradually decreases towards its periphery;
- in *A. niger* deleted for FlbA (displaying a nonsporulating, slow-growing, and autolytic phenotype), *anafp* expression is strongly upregulated (5-fold). Note that FlbA is conserved in aspergilli and known to stop vegetative growth during the process of conidiation. Its main function is to activate the transcription factor BrlA in response to the extracellular signaling molecule FluG. BrlA, in turn, is the central regulator of asexual development in aspergilli [43];
- in *A. niger* deleted for BrlA (displaying a nonsporulating, slow-growing, but nonautolytic phenotype), *anafp* expression is upregulated as well (2-fold). However, induction of *anafp* expression precedes *brlA* expression in the wild type; that is, BrlA cannot be the first regulator of *anafp*;
- *anafp* expression is not induced upon cell wall stress (provoked by caspofungin, fenpropimorph, FK506, aureobasidin A, natamycin), secretion stress (induced by DTT, tunicamycin), or confrontation with *Bacillus subtilis*;
- *anafp* is not important for polar growth of *A. niger*, as morphology mutants (TORC2, RacA) do not show altered *anafp* expression;
- although AnAFP is a secreted protein, it becomes detectable in culture supernatant only at 140 h post-carbon depletion in the Δ*flbA* strain, although its transcription peaked at 16 h post-carbon depletion in wild-type (N402), Δ*flbA*, and Δ*brlA* strains [44]. Similarly, a number of hydrolytic genes that displayed strong transcriptional upregulation during carbon starvation, including chitinases and mannanases, were not detectable in culture supernatants;
- the *anafp* promoter is activated during osmotic stress provoked by different salts including NaCl, CaCl_2_, KCl, MgCl_2_, and KH_2_PO_4_;
- the *anafp* promoter is activated in the presence of H_2_O_2_, but inhibited in the presence of menadione. Such an opposing effect of both oxidants is in good agreement with previous findings that autophagy-deficient *A. niger* strains deleted for *atg1* (predicted Ser/Thr kinase) or *atg8* (predicted autophagy-related ubiquitin modifier) are both more sensitive to H_2_O_2_, but less susceptible to menadione when compared to the wild-type strain [45].

A co-expression network analysis using data from all 155 different cultivation conditions and calculated with a very stringent Spearman’s rank correlation coefficient uncovered that 605 (381) of *A. niger* genes show a positive (negative) correlation with *anafp* expression. Gene ontology enrichment analyses revealed that the processes positively correlated with *anafp* expression belong to development, cellular polysaccharide catabolism, antioxidant activity, and *O*-glycosyl hydrolase activity, whereas processes negatively correlated with *anafp* expression include translation as well as amino acid, nucleobase, and pigment biosynthesis [10]. Among positively correlated genes, worth emphasizing are autophagy-related ones (orthologs of *S. cerevisiae* Atg4, Atg8, and *A. nidulans* metacaspase CasA). The network analysis also predicted that at least seven transcription factors control *anafp* expression [10], three of which are well-studied regulators in other aspergilli: CreA (carbon catabolite repressor, [46]), StuA, and VelC (regulators of asexual development and secondary metabolism [47,48,49,50]). All three transcription factors were experimentally proven to modulate *anafp* promoter activity, with CreA and StuA being strong repressors ([10] and [51]).

### 3.2. When and Where Is the anafp Gene Expressed?

In the course of the experiments mentioned above, we noticed that generating an *A. niger* Δ*stuA* deletion strain in a wild-type background results in only a few, severely sick transformants, which is not the case in a Δ*stuA*Δ*anafp* double deletion background [51]. This observation suggests that premature and strong overexpression of the *anafp* gene due to *stuA* deletion is highly detrimental for *A. niger*. Likewise, constitutive expression of AFPB in *P. digitatum* resulted in reduced growth [37]. These data indicate that expression of antifungal proteins is under tight control. Figure 2 depicts the expression profiles of the *afp* and *anafp* genes over time in *A. giganteus* and *A. niger*, respectively, visualized with appropriate reporter strains. Obviously, there is only a limited time window during growth and development of both *A. niger* and *A. giganteus* where *afp* and *anafp* become expressed. We propose that this is because the encoded AFPs fulfill an important function for their hosts only during this specific period. Outside this time window, gene expression of both genes is repressed to negligible levels.

Surprisingly, and in agreement with data for the *afpB* gene in *P. digitatum* [37], deletion of *anafp* in an *A. niger* wild-type background does not provoke any detectable phenotype when the mutant is cultivated on agar plates or under submerged conditions in shake flask cultures [10]. Neither germination rate nor sporulation efficiency were affected by the deletion. Likewise, biomass accumulation was not affected in the Δ*anafp* strain [10]. Absence of any *anafp*/*afpB* deletion phenotypes suggests that their phenotype is very subtle or that other redundant proteins could take over AFP function. Still, discrepancies with the *paf* gene from *P. chrysogenum* are observed, where deletion of the gene results in markedly reduced sporulation [29].

**Figure 3:**
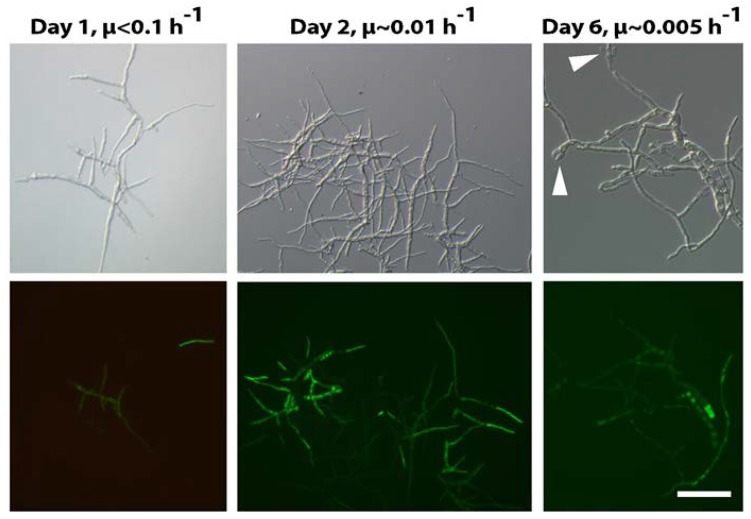
Morphological differentiation of *A. niger* during substrate-limited growth in retentostat cultures as visualized by DIC (differential interference contrast) microscopy (upper panel) and fluorescence microscopy (bottom panel). Mycelium of an P*anafp::eyfp* reporter strain after 1 day (μ < 0.1 h^−1^), 2 days (μ ~ 0.01 h^−1^), and 6 days (μ ~ 0.005 h^−1^). Fluorescence represents the activated *anafp* promoter and is only visible in individual compartments. Note that after day 6, newly formed spores become visible (arrows). Picture is taken from [10], licensed under CC-BY 4.0. μ: growth rate. Bar = 20 µm.

Remarkably, time-dependent regulation of the *anafp* promoter does not occur homogeneously in all cellular compartments of *A. niger* mycelium. This is evident in a fluorescently-labeled reporter strain in which the *anafp* ORF (open reading frame) was replaced with the *eyfp* reporter gene [10]. Under severe carbon and energy limitation (achieved in a controlled manner in bioreactor retentostat cultivations), YFP fluorescence could only be detected in individual compartments. As depicted in Figure 3, the *anafp* promoter is only active in older mycelia with strongly vacuolated compartments or in newly formed mycelium displaying very thin hyphae. We recently showed that high vacuolization in *A. niger* mycelial compartments is indicative of autophagy and precedes it; that is, drives cryptic growth of thin hyphae [45,52]. It is thus tempting to speculate that AnAFP has a role during this process. Newly formed spores do not show any *anafp::eyfp* expression.

Taken together, transcription of the *anafp* gene seems to be under the highest temporal and spatial control. Its expression profile is concomitant with the expression profile of early starvation response genes functioning in nutrient mobilization and autophagy during developmental processes. The gene-correlation network predicted that its function is somehow connected to autophagy-related processes and uncovered three nutritional and asexual developmental transcription factors controlling *anafp* expression (CreA and StuA: repressors, and VelC: activator), which we could verify in vivo using a reporter system ([10] and manuscript in preparation). Its expression not only parallels the expression of autophagic proteins, but is selectively activated in highly vacuolated compartments of the vegetative mycelium which are supposed to undergo autophagy for nutrient recycling. In view of the membrane activity of AnAFP and its subtle induction of cell wall stress in *A. niger*, we propose that the tight spatial and temporal control of its gene expression enables AnAFP to fulfill an important function for its producing host during nutrient starvation; that is, during autophagic processes. With this activity, AnAFP thus contributes to the survival of *A. niger*. This hypothesis is supported by publications from others who demonstrated that asexual sporulation of *A. nidulans* is accompanied by autolytic and apoptotic processes [53,54,55,56]. It is also in agreement with the observation that there is a considerable delay between induction of *anafp* gene expression and detection of AnAFP in the culture supernatant of *A. niger* [44,57]. Some AFPs (e.g., PAFB from *P. chrysogenum* and AFPB from *P. digitatum*) even remained undetectable in the medium although their encoding genes were transcribed at high levels [13,37]. It cannot be excluded that the proteins might have escaped detection; however, it is conceivable that AFPs firstly localize at the cell wall or inside the cell before being released into the medium at later growth stages. The only AFP family member for which an intrinsic function has been demonstrated so far is the PAF protein from *P. chrysogenum*. It was observed that apoptosis rates and expression levels of autophagic genes are lowered during carbon starvation in a strain lacking a functional *paf* gene. Also, reduced sporulation was observed in the *paf* deletion strain, providing for the first time indirect evidence that the PAF peptide is important for the process of asexual sporulation in *P. chrysogenum* [58].

## 4. A Small Interlude: Sporulation in Bacteria and the Importance of Cannibal Toxins

Cannibalism is a phenomenon occurring during early stages of sporulation of the Gram-positive bacterium *Bacillus subtilis* and involves the production of toxins of a sporulating subpopulation killing genetically identical but nonsporulating sibling cells [59]. In brief, endospore formation in *Bacillus* is considered as a last-resort [60] or bet-hedging (i.e., risk-spreading) strategy [61] under starvation conditions to ensure survival of the species. It is a process that takes about 8–10 h and results in the formation of endospores resistant to UV stress, chemical stress, and heat [60]. The killing factors (“cannibal toxins”) are the sporulation killing factor Skf, a 26-amino-acid-long ribosomally synthesized and post-translationally modified cyclic sactipeptide, and the sporulation delaying protein Sdp, a 42-amino-acid-long ribosomally synthesized peptide [62]. Both are secreted and lyse nonsporulating sibling cells that have not developed immunity to them. Immunity is conferred on the one hand by expression of an ABC (ATP binding cassette) transporter that exports Skf out of the cell, thereby avoiding death of the producing cell, and on the other hand, by expression of the integral membrane protein SdpI, which acts as signal transduction protein and sequesters a repressor protein (for details, see [59]). About two-thirds of *B. subtilis* cells in a sporulating population eventually become killed by both killing factors [59].

Interestingly, *skf* and *sdp* mutants of *B. subtilis* do not lose the ability to sporulate. In contrast, the sporulation process is accelerated in these strains, demonstrating that wild-type cells try to delay the commitment to sporulation via production of both cannibal toxins. It is generally thought that delaying sporulation as long as possible is of advantage for *B. subtilis* because (i) sporulation as a developmental process is energetically costly, (ii) spores resume growth not as fast as vegetative cells when nutrients are available again, and thus (iii) sporulation confers an ecological disadvantage relative to cohabitating microorganisms [59]. The master regulator of the initiation of sporulation is Spo0A, which controls expression of about 120 genes [63]. Spo0A-responsive genes fall into two categories: those responding to low concentrations of Spo0A (because of having high-affinity bindings sites in their promoters) or to high concentrations (because of low-affinity bindings sites in their promoters). Operons involved in cannibalism (production of Skf and Sdp) as well as multicellular aerial structures (in which sporulation will take place in natural isolates; this trait has been lost during domestication of *B. subtilis* [64]) belong to the first category. Cannibalism and formation of aerial structures are thus considered as “a prelude to spore formation” [59]. If environmental conditions still favor sporulation, Spo0A is increasingly expressed, and cells are committed to sporulation and express genes important for spore formation [59].

Cannibal toxins are also active against other bacteria; for example, Skf inhibits growth of *Xanthomonas oryzae* [65] and *Escherichia coli* [66], and Sdp is known to kill *Staphylococcus aureus* and *S. epidermidis* at IC_50_ (half maximal inhibitory concentration) values similar to vancomycin [62]. This suggests that *B. subtilis* cannibal toxins also participate in defensive or predatory behavior directed at other species [59]. Purified Sdp was indeed shown to act as endogenously produced Sdp (delaying sporulation) and to collapse the proton motive force in Gram-positive species (*B. subtilis*, *S. aureus*, *S. epidermidis*) as well as in a gram-negative *E. coli* mutant strain with a compromised outer membrane. Loss of proton motive force induced autolysin-mediated lysis of the cells, demonstrating that a cannibalistic toxin can also be a defensive toxin. This also highlights an interesting survival strategy: Cannibal toxins induce an autolytic program in neighboring cells irrespective of whether they are from the same or from a different species. Affected cells cannot escape by simply moving away; instead, their fate is determined: autolysis is induced, and lysed cells provide nutrients to immune cells to promote their own growth [67].

## 5. Are AFPs Cannibal Toxins?

As discussed above, cannibalism is a phenomenon occurring during early stages of bacterial sporulation and aims to delay commitment to this process. We will show in the following that conceptual similarities exist to the process of asexual sporulation in filamentous fungi and propose that AFPs could, similarly to Skf and Sdp, function as cannibal toxins in ascomycetes. No experimental evidence is available so far for this hypothesis; however, parallels exist between the processes of sporulation at the heuristic level of decision-making processes and at mechanistic levels regarding the time- and space-dependent regulation of gene expression and mode of action of these antimicrobial peptides.

Developmental transition from vegetative growth to asexual sporulation (conidiation) in the fungal class of ascomycetes is best studied in the model *A. nidulans*. Several negative key regulators (SfgA, VosA, and NsdD) inhibit precocious commitment to the formation of asexual spores, thereby allowing growth of vegetative hyphae as long as sufficient carbon sources are available [68]. Acquisition of developmental competence thus involves elimination of negative regulation and will be briefly summarized as follows (for more details, the reader is directed to [68]): The essential activator for conidiation is the transcription factor BrlA, which is expressed in response to the developmental FluG signal (a diorcinol–dehydroaustinol adduct [69]). BrlA in turn induces the transcription factor AbaA, which in turn activates the transcription factor WetA. All three regulators thus constitute a central regulatory hub that positively controls gene activation during conidiophore development and spore formation [35]. A genetic cascade upstream of BrlA–AbaA–WetA important to activate BrlA is the FluG–Flb cascade [70,71,72]. This cascade is controlled by the negative regulator SfgA, which acts between FluG and Flb proteins [73]. Note that inactivation of FluG or FlbA results in the absence of conidiation [74]. FlbA is a regulator of G-protein signaling (RGS) and arrests vegetative growth during conidiation by activating BrlA in response to FluG [43]. Note that *anafp* expression in *A. niger* takes place between expression of *flbA* and *brlA*; hence BrlA cannot be a regulator of *anafp* [10].

Do AFPs from ascomycetes play any role in asexual sporulation similarly to those of cannibal toxins during bacterial sporulation? We do not know yet. However, considering the data accumulated so far for AFP, AnAFP, and PAF (for details, see Section 3) and the Darwinian assumption that biological functions differ “in form but not in kind”, we provide here arguments for a functional relationship between bacterial cannibal toxins and fungal AFPs:

- expression of these peptides is strongly repressed during vegetative growth;
- expression of these peptides is derepressed during environmental stress conditions favoring sporulation;
- expression of these peptides is under tight temporal and spatial control;
- overexpression of these peptides is detrimental for the producing strain and causes autolysis;
- expression of *anafp*, similarly to those of *skf* and *sdp* operons, occurs only in a subpopulation of cells;
- expression of these peptides is concomitant with expression of autophagic and autolytic proteins;
- expression of these peptides decreases when commitment to sporulation has been achieved;
- deletion of the respective peptide-encoding genes does not prevent sporulation, but might affect timing and/or efficacy of sporulation;
- sporulation causes death of a significant portion of the population, which releases nutrients to feed survivors;
- host cells are immune against their own toxins; that is, in MIC assays, they appear less sensitive to their own AMPs compared to alien AMPs;
- these peptides are membrane-interacting, whereby Skf and Sdp act specifically antibacterial and AFPs specifically antifungal;
- the primary function of these peptides could be to kill genetically identical siblings ( cannibalism); however, they can also function in the defense against other fungal (or bacterial) species.

Several experimental approaches will be necessary to answer the question as to whether AFPs indeed act as cannibal toxins. Clearly, single-cell analytical approaches with high temporal resolution allowing detailed analysis of transcriptional and metabolic heterogeneity in mycelial cultures or colonies are required. Combining single-cell analytics with controlled deregulation of *afp* genes in wild-type and mutant backgrounds impaired in autophagic/autolytic processes will provide unique opportunities to resolve metabolic heterogeneity in mycelial populations and to verify the hypothesis proposed in this review. From an evolutionary point of view, it will be interesting to study further AFP orthologs from other ascomycetes to increase our understanding of programmed cell death in lower eukaryotes (note that AFP-encoding genes have so far not been identified in genomes of basidiomycetes, perhaps because they are not capable of asexual sporulation). Not only are cannibal toxins, such as Sdp and Skf of *B. subtilis*, known to induce programmed cell death; cannibalism is also prevalent in cancer cells, where neighboring cells become ingested upon carbon starvation, a process called entosis [75,76]. Cannibalism could thus be a conserved cellular response in prokaryotes and eukaryotes enabling cell survival through nutrient recycling from lysed neighbor cells.

## 6. Conclusions

Characterization of the antifungal peptides AFP and PAF has provided considerable biological understanding of processes underlying their antifungal activity, including genetic susceptibility factors, cell wall composition/remodeling enzymes, and signaling components involved in their toxicity. However, knowledge surrounding the gene regulation of members of the AFP family and the puzzling link to asexual developmental processes is severely limited. The so-far available data are descriptive, and mechanistic explanations of their temporal and spatial regulation are completely absent. We provide here a conceptual framework for the mode of action of AFPs that goes far beyond their antifungal activity, which is in agreement with accumulating evidence suggesting that AMPs are likely multifunctional [77,78]. Several hallmarks of *afp*, *anafp*, and *paf* gene expression during asexual sporulation of *A. giganteus*, *A. niger*, and *P. chrysogenum*, respectively, parallel hallmarks of cannibal toxin expression and function during sporulation of *B. subtilis*. We have summarized these and provided plausible arguments that members of the AFP family could indeed act as cannibal toxins in fungi. We believe that the knowledge gap regarding cellular functions of AFPs can be filled by learning from bacteria capable of asexual sporulation. The aim of our review is to stimulate fungal (and bacterial) scientists to think about their model organisms and model proteins in a broader context.

A question arising is whether AFPs act as sensor, signaling, or effector molecules, leading to intracellular destabilization of plasma membranes and subsequent cell lysis. Given a recursive relationship such as in the immunobiology of higher eukaryotes [10], AFPs could be effector molecules activating their own sensor/signaling molecules in order to provoke a strong defense response. Hence, it is conceivable that all of these options are true. All technological requirements and molecular tools ranging from single-cell analytics and comparative genomics to targeted gene (in)activation are available and better than they have ever been to study these possibilities. The ultimate goal is to understand the molecular mechanisms behind AFP-related processes at the intersection of cell function and dysfunction, cell survival, and death. This knowledge will help us to identify the “Achilles’ heel” of filamentous fungi and thus new excellent drug target(s) for novel antifungal agents and strategies. Such an understanding will also assist in identifying new leads for improved growth of the industrial cell factories *A. niger* and *P. chrysogenum* during carbon starvation, which is frequently encountered during industrial fermentation processes.
